# A randomized trial testing the effectiveness of virtual reality as a tool for pro-environmental dietary change

**DOI:** 10.1038/s41598-022-18241-5

**Published:** 2022-08-22

**Authors:** Adéla Plechatá, Thomas Morton, Federico J. A. Perez-Cueto, Guido Makransky

**Affiliations:** 1 Department of Psychology, University of Copenhagen, Øster Farimagsgades 2A, 1353 Copenhagen K, Denmark; 2 Department of Food, Nutrition and Culinary Science, Umeå University, Lärarutbildningshuset, Plan 4, 901 87 Umeå, Sweden

**Keywords:** Psychology, Human behaviour

## Abstract

This study investigates the impact of an efficacy-focused virtual reality (VR) intervention designed according to instructional design principles on eating behavior. In the preregistered intervention study, psychology students were randomly assigned to nine seminar blocks. Employing parallel design, they were allocated to either a VR intervention to experience the environmental impact of food behavior (1) and alter the future by revising food choices (2) or to a passive control condition. The data from 123 participants (78% female, mean age 25.03, *SD* = 6.4) were analyzed to investigate the effect of the VR intervention on dietary footprint measured from 1 week before to 1 week after the intervention. The VR intervention decreased individual dietary footprints (*d* = 0.4) significantly more than the control condition. Similarly, the VR condition increased response efficacy and knowledge to a larger extent compared to the control. For knowledge, the effect persisted for 1 week. The VR intervention had no impact on intentions, self-efficacy, or psychological distance. Additional manipulation of normative feedback enhanced self-efficacy; however, manipulation of geographical framing did not influence psychological distance. This research received no financial support from any funding agency and was registered on 15/09/2021 at Open Science Foundation with the number 10.17605/OSF.IO/2AXF3.

## Introduction

Emerging evidence suggests that immersive Virtual Reality (VR) can be a powerful tool to promote pro-environmental behavior^1^. For example, VR-mediated natural experiences have been shown to increase connectedness to nature^2,3^ and to promote pro-environmental behavior as effectively as a real-life nature hiking experience^4^. Immersive VR enables the creation of realistic and embodied situations which provide rich sensory input^5^, ensuring that virtual experiences nonetheless feel proximal to the individual^6^. According to some^7,8^, the experience of proximity to the self is central to action against climate change—the consequences of which often remain too abstract, or too spatially and temporally distant, to trigger risk perception and readiness to act. Nevertheless, low-risk perception is not the only barrier to action: heightening risk perceptions can also trigger defensiveness in the face of threat^9–11^. One important determinant of whether people respond adaptively or maladaptively to perceived risks and feelings of threat is the efficacy they perceive in taking action. Consistent with this, both perceived self-efficacy (the belief in the personal capacity to execute desired actions) and response-efficacy (the belief that the actions will contribute to the solution) have also been identified as important drivers of pro-environmental behavior^12^. Interventions that simultaneously induce risk perceptions and build up efficacy beliefs should be maximally effective, both in general^13^ and in the specific context of attempts to promote climate-friendly diets^7^.

VR has a number of features that dovetail nicely with the identified psychological features of climate action. A recent theory of immersive learning^8^ describes how the dual affordances of presence (the feeling of being physically and socially present in the virtual environment^14^), and agency (the sense of being in control of one’s actions in the virtual environment^15^), make VR a potentially effective tool for learning and behavioral change. Both presence and agency are especially high in interactive simulations experienced through head-mounted displays (HMDs)^16^ and can lead to enhanced motivation, self-efficacy, and response efficacy (factors crucial for eliciting behavioral change^8,17^) when VR simulations are developed according to instructional design principles^8,15,18,19^. Consistent with this model, recent research shows that VR provides mastery experiences that positively impact self-efficacy and response-efficacy beliefs^17,20^, and that VR can help individuals to visualize the impact of their behavior on the natural environment^21^.

Despite the considerable potential of reducing GHGs emissions by adopting more plant-based diets^22,23^, not many VR studies investigated the potential of VR to facilitate a switch to more sustainable diets. One recent study by Meijers et al.^21^ reports the influence of climate impact messages on behavior in a virtual supermarket, and the indirect effect of these on self-reported consumer decision-making 1 week after the experiment. Additionally, Fonseca and Kraus^24^ reported preliminary evidence that VR can be a suitable tool for promoting change in sustainable dietary behavior. According to their results, an immersive VR video resulted in a larger preference for vegetarian meals immediately after the intervention compared to a control group, and this was also reflected in spontaneous choices of vegetarian over non-vegetarian snacks after the simulation.

Outside of the VR field, environmental research shows that social norms (alongside self-efficacy, response efficacy, and negative emotions), play a crucial role in regulating climate-related behavior^12^. Properly administered normative feedback (i.e., about one’s performance relative to significant others) has been demonstrated to be useful for motivating behavior change^25^, and for supporting individual feelings of self-efficacy^26^, across a variety of domains, including the environment. Furthermore, the perception that climate change is distant from the individual—that is, happening to people who are far away from both in time and space—is often argued to be the leading barrier to climate change inaction^7,27^. Yet, despite this common assumption research manipulating psychological distance has produced mixed findings^28–30^, suggesting that this approach can only have limited utility within interventions designed to encourage climate-related action. Because immersive VR allows realistic visualization of climate change on nature at different geographical and temporal scales, it seems to be a perfect tool for further investigating the role of psychological distance in motivating action. For example, a previous study conducted using non-immersive VR showed that navigating a polluted river framed as geographically closer resulted in higher risk perception, which, together with self-efficacy, predicted self-reported pro-environmental behavior^31^.

Despite the emerging evidence that VR can promote pro-environmental intentions and, in some cases, behavior, there is only limited evidence about the ability to facilitate the switch to plant-based diets^21,24^. Although the previous study mentioned above^21^ identified personal response efficacy as an important mechanism for change in response to VR, immediate behavior change was not separate from the manipulation of impact messages, and delayed behavior change was self-report and not focused on the actual environmental impact. Furthermore, the VR study^24^ using emotional VR videos focused on reducing meat consumption reported only the effect immediately after the intervention. As such, consequences for reductions in individual carbon footprints and the long-term impacts of the VR interventions are still unknown and have not been tasted with larger randomized controlled trials.

Furthermore, the VR research applying methods from environmental research, like manipulating psychological distance or normative feedback, in vivid, immersive environments to elicit PEB is scarce. Some previous VR studies indicate that VR would be suitable for bridging the psychological distance of climate change using the spatial reference^31,32^ or level of immersion^32^. Nevertheless, the role of distance framing or normative feedback on eating behavior using an efficacy-focused VR intervention has not been investigated before.

In order to investigate the above-mentioned knowledge gaps, here we tested the behavioral consequences of a novel VR intervention designed to promote self-efficacy and response-efficacy beliefs. The intervention was designed using instructional design principles (e.g., embodiment, personalization, and modality principle) to facilitate the impact of agency and presence on the behavioral outcome^8,18^. Furthermore, similar to previous studies, we aimed to increase response-efficacy by visualizing the impact of individual behavior^21,33,34,35,36^, in our case the impact of food choices on the natural environment. As previous VR research shows the importance of gradual changes^37^ and vivid messages^38^ for behavioral change, the impact of current food choices on the environment is visualized continually by transporting participants 30 years into the future. Furthermore, the educational part of the intervention provided users with explicit instruction on how to behave environmentally as some research highlights how the lack of specific guidelines can limit transfer to real-life behavior^39^ and actual impact in terms of carbon emissions^21^. Finally, to build self-efficacy using mastery experience^40,35^, users were allowed to change their behavior and were given customized feedback about their choices through the gradual restoration of nature.

In this study, we compare the effectiveness of this VR intervention against a passive control condition. As stated in the study plan that was preregistered before the data collection commencement, we hypothesized that:

### H1

The VR intervention will lead to a larger decrease in the carbon footprint compared to the control group.

### H2A–E

The VR intervention will impact all predictors (intentions, self-efficacy, response-efficacy, knowledge, psychological distance) of pro-environmental behavior to a larger extent compared to the control group.

### H3A–D

In the follow-up, the VR intervention will impact all predictors (self-efficacy, response-efficacy, knowledge, psychological distance) of pro-environmental behavior to a larger extent compared to the control group.

Furthermore, we explored if the VR simulations can be enhanced by using other interventions that have been tested within environmental research: normative feedback and geographical location (distant vs. proximal).

## Methods

This study’s design, data collection, and analysis plan of this randomized control trial were registered at Open Science Foundation on 15/09/2021, prior to data collection with the number 10.17605/OSF.IO/2AXF3. Unless otherwise stated, the presented study followed the preregistered plan (access here https://bit.ly/34hFpT4). The study was approved by the Institutional Review Board at the Psychology Department, University of Copenhagen, approval number IP-IRB/02092021, and was performed in accordance with the ethical standards of the Declaration of Helsinki (1964) and its subsequent amendments. Informed consent was obtained from all participants.

### Participants

Participants were recruited from an Educational psychology course at a large Scandinavian university and tested between September 16 and October 7, 2021. The participants who completed the post questionnaire (*N* = 123) were used for the relevant analyses (Hypothesis 2). For the analyses of food behavior and the long-term impact of the intervention (Hypotheses 1 and 3), we used only participants who completed the whole procedure (*N* = 90). As the sample size was determined by the number of students willing to participate in the experiment, we calculated the minimum detectable effect size (MDES) for the reached sample size. The MDES was *f *^2^ = 0.11 for the analyses of the follow-up data stated in the preregistration (Hypotheses 1 and 3) and *f *^2^ = 0.08 for Hypothesis 2. These values correspond to small effects^41^; thus, our sample size for the main analyses was considered sufficient for detecting desired effects. Table 1 contains the baseline characteristics of the groups.

**Table 1:** Baseline characteristics of the analyzed sample. The continuous variables are summarized as the mean (standard deviation).

Sample characteristics	Control *N* = 56	VR *N* = 67
Age	24.9 (6.50)	25.1 (6.36)
% Female	82%	75%
% Omnivorous diet	59%	66%
Previous VR experience (median)	Never	Never
Preexisting knowledge	3.70 (1.28)	3.79 (1.44)
Pretest self-efficacy	4.04 (0.69)	4.20 (0.75)
Pretest response-efficacy	3.54 (0.86)	3.52 (0.80)
Pretest psychological distance	5.09 (2.13)	5.03 (2.04)
Pretest dietary carbon footprint	118 (64.9)	124 (82.2)

### Study design

Prior to the study commencement, participants were randomly assigned to nine seminar groups of equal sample size by the faculty administrators (not study administrators). We consequently randomly assigned four of these groups to the VR condition and five groups to the control condition. Participants were not aware of the assignment to different intervention groups. Additional simple randomization (random numbers) was implemented in the VR intervention condition to assign participants to the specific VR intervention.

The trial followed a parallel design, with between-subjects repeated measures (pre; post; follow-up) with VR intervention as the experimental condition and the passive group as the control condition. Participants filled out the pretest questionnaires on their personal smartphones or computers using SurveyMonkey 1 week before the experiment, immediately after the intervention, and 1 week after the intervention. Participants in the control condition did not receive any intervention but were given the VR intervention after the data collection was completed, not disadvantaging any students from the course. To investigate the potential impact of different design principles, we administered the VR intervention in 2 × 2 design varying geographical location (distant vs. proximal) and normative feedback (generic vs. normative feedback), which resulted in four experimental conditions.

**Figure 1:**
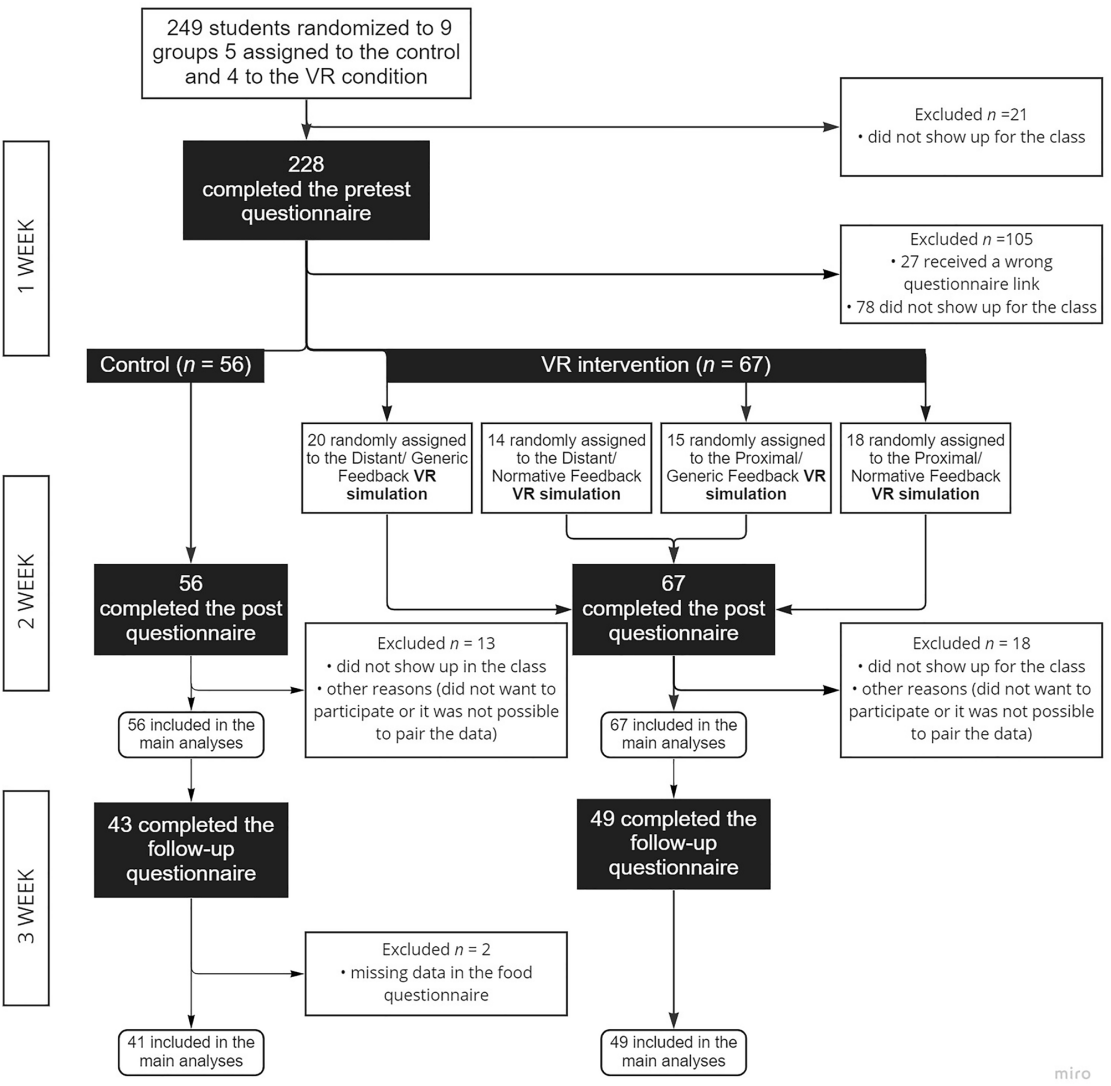
Trial profile shows participants’ flow into intervention arms and analyses.

Participants took part in the experiment over the course of 3 weeks during their Educational psychology class. In the first week, participants completed the questionnaire in their classroom. In the second week, participants in the VR intervention condition were randomly assigned to either distant or proximal geographical location and to general or the normative feedback condition. They completed the VR simulation using Oculus Quest 1 or 2 distributed evenly across the conditions in two different laboratories. The intervention lasted approximately 15 min. Immediately after the VR intervention, they were asked to complete the post questionnaire. In the control condition, participants were asked to fill out the post questionnaire during their class in the second week of the experiment. Finally, in the third week, all the participants completed the questionnaire in the classroom. The participants’ flow is depicted in Fig. 1, and the complete protocol is available in the Supplementary Material and online https://bit.ly/3C6ZlEO. In the section below, we describe the VR intervention and its variants.

### Intervention

#### VR simulation and conditions

**Figure 2:**
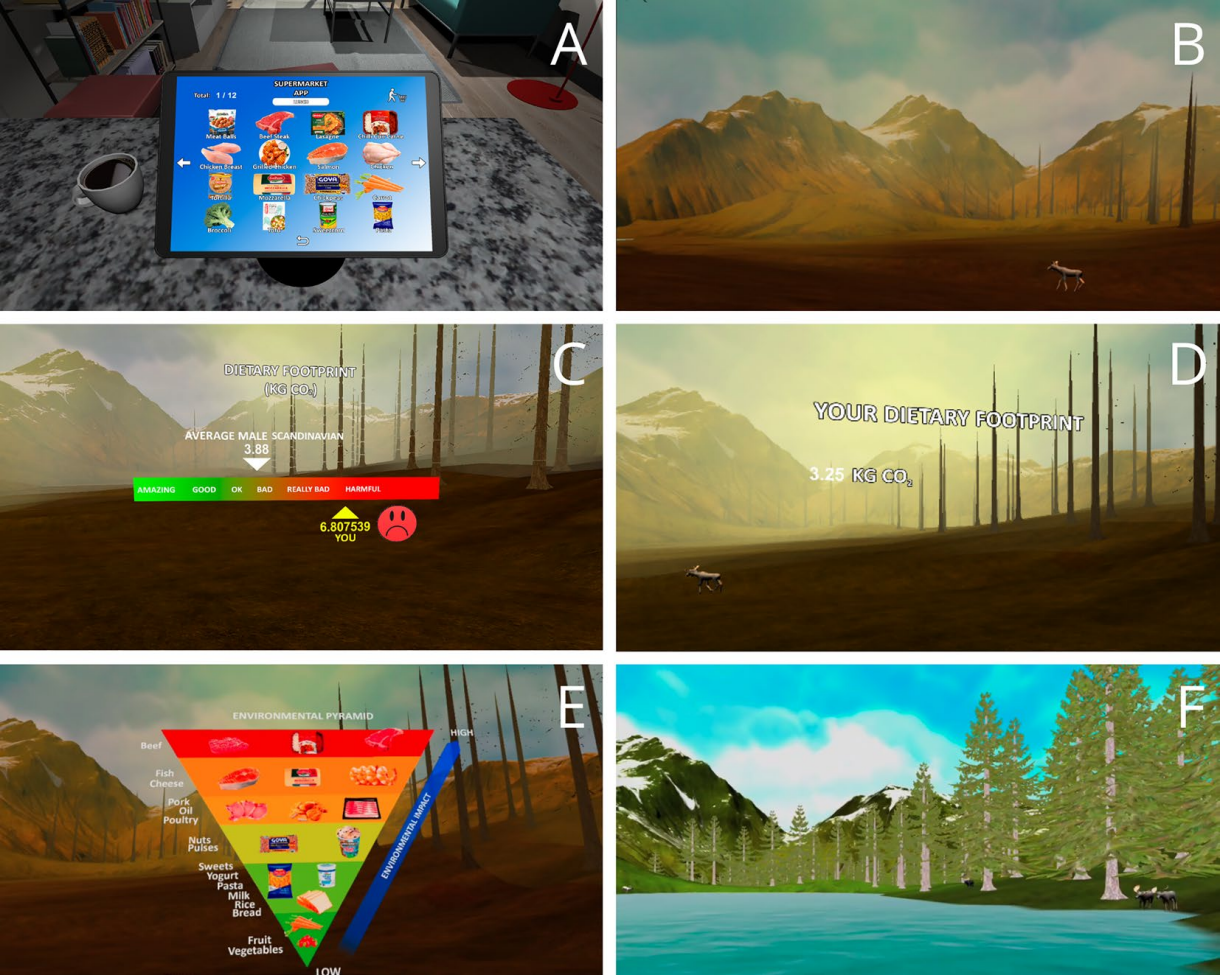
Virtual reality intervention started in a living room where participants indicated they preferred food choices (**A**) and then they virtually traveled to a national park where they witnessed the consequences of climate change (**B**). Thereafter, according to the condition, participants either received normative feedback about their dietary carbon footprint compared to the average (**C**) or just generic feedback (**D**). Consequently, all the participants were educated about the environmental impact of foods (**E**) and selected new foods, and experienced restoration of the environment if they chose foods with a low environmental footprint (**F**).

Phase 1 of the VR intervention started with an online shopping simulation in which participants selected the foods they typically purchase for lunch, breakfast, dinner, and snacks (depicted in Fig. 2A). In Phase 2, the participant was transported to a natural park. The park’s stated geographical location depended on the condition to be either distant (Rocky Mountains, USA) or proximal (Sonfjället National Park, Sweden). The choice of these two locations was based on their plausible visual similarity—both the Rocky Mountains and Sonfjället in Sweden are open, mountainous landscapes with similar flora and fauna—and their difference in distance from individual participants (who were Scandinavian, and therefore closer to Sweden than the USA). In both locations, the simulation transported participants 30 years into the future, so that they could witness the gradual degradation of this natural environment as a consequence of the current food-related GHG emissions (see Fig. 2B). Based on their own selection in Phase 1 (i.e., their food choices), the agent informed them about their current dietary carbon footprint. This information contained either normative feedback (i.e., was in comparison with other Scandinavians: Fig. 2C) or was generic (i.e., provided information about emissions without any comparison: Fig. 2D). The dietary carbon footprint was calculated using values reported by the CONCITO climate database^42^ multiplied by the standard portion. Within the simulation, a pedagogical agent in the form of a park ranger educated the participant about the environmental consequences of the specific foods (see Fig. 2E)^42,43^. In the final phase (Phase 3), participants again selected the food in the shopping simulation and were instructed to choose the food with lower impact (the categories were highlighted). According to their new choices, the natural environment changed (according to 10 possible levels) based on what would happen if everyone adopted the same diet (see Fig. 2F).

#### Control condition

Participants in the control condition filled out the questionnaires in the three consecutive weeks (pretest, posttest, follow-up) without any intervention and without receiving information about the condition they were allocated in. The participants in the control condition experienced VR intervention after completion of the data collection.

### Outcome measures

The primary outcome variable was the change in *dietary carbon footprint* calculated from the results of the food frequency questionnaire administered 1 week before and 1 week after the intervention. The average carbon footprint for each category^42^ was multiplied by the frequency indicated by the respondents and pooled into the general dietary footprint.

*Self-efficacy* was measured with two items adapted from Huang et al.^44^ and two items focused on eating behavior^35^. The two items from Huang et al.^44^ were later considered to be too general to measure the self-efficacy concept, leading to deviation from the preregistration plan as only the two items were used for the following analyses. Nevertheless, the exclusion of the two items did not influence the conclusions of this study. We report the results of the analyses including all four self-efficacy items in the Supplementary Material. Three items measuring *response efficacy*, and four items measuring *behavioral intentions* were adapted from Hunter and Röös^7^. *Psychological distance* was assessed using five items adopted by Spence et al.^7^. *Knowledge* was measured by asking participants to indicate the level of emissions for 15 foods presented in the simulation*.* Self-efficacy, response efficacy, and psychological distance were measured 1 week before, immediately after, and 1 week after the intervention*.* Knowledge was measured immediately after and 1 week after the intervention.

Finally, a one-item *preexisting knowledge* variable (seven-point scale from Very low to Very high) and reported dietary lifestyle were used as a covariate in the analyses. The Supplementary Material details the wording of all measures collected in the study.

### Statistical analysis

Statistical analyses were performed using R^45^. Linear regression models were used to investigate the impact of the intervention on the outcome variables postscores and follow-up scores adjusted for pretest scores. The regression models investigating the impact of different VR conditions on the posttest outcome variables were adjusted for pretest scores and the relevant condition. For the outcomes measured only in posttest and follow-up (intentions and knowledge), we used dietary lifestyle and preexisting knowledge as covariates respectively.

## Results

### Changes in dietary footprint 1 week after the intervention

**Figure 3:**
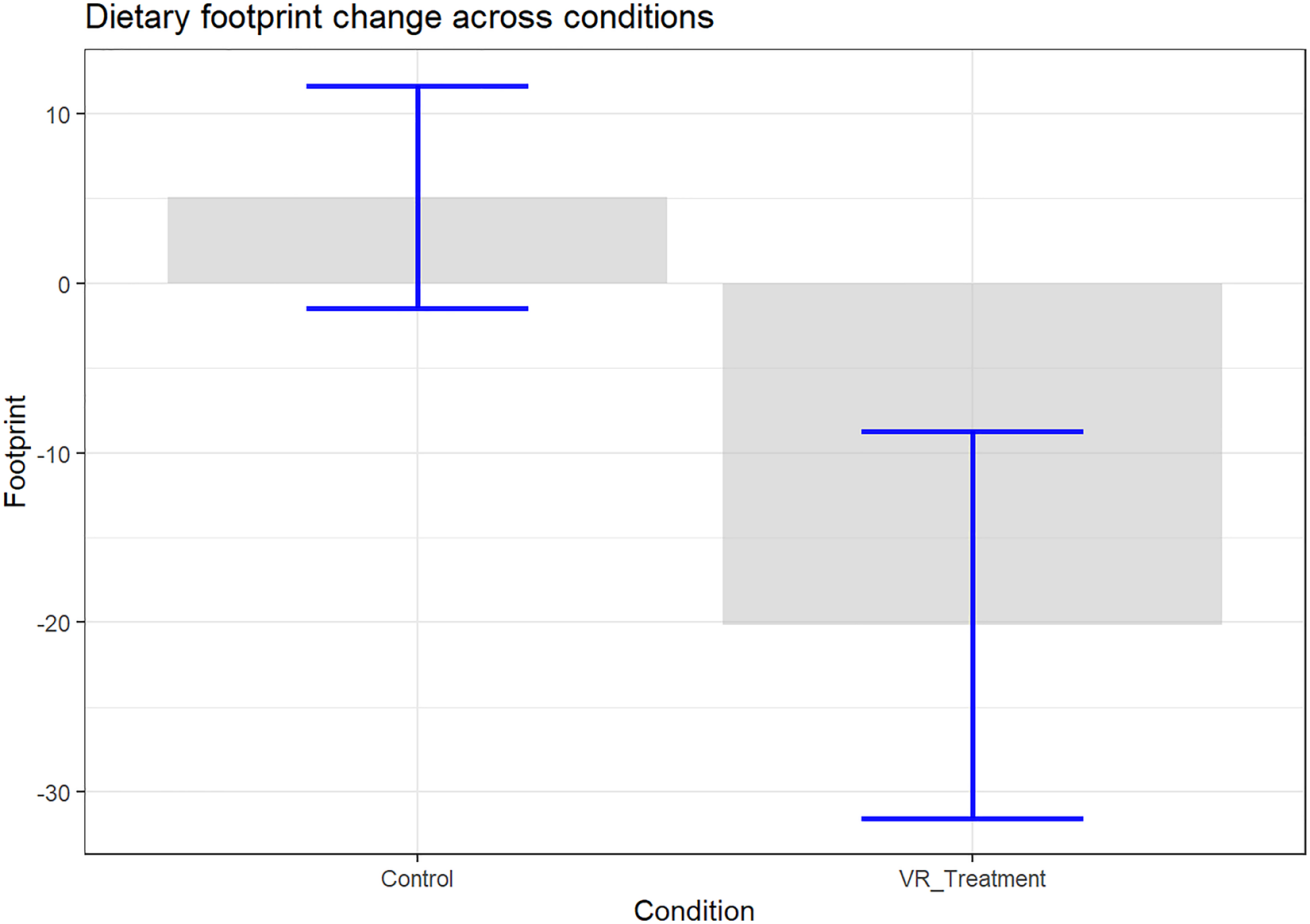
Change in dietary carbon footprint from pretest measurement to the 1-week follow-up for both conditions. Negative values indicate a decrease in dietary carbon footprint. Error bars are 95% confidence intervals.

To investigate if the VR intervention has the potential to facilitate the switch towards a more sustainable diet, we compared the VR intervention and control condition’s effects on dietary carbon footprint. The average change in dietary footprint for the VR and control conditions from pretest to 1-week follow-up is depicted in Fig. 3.

Figure 3 shows that there was a significant difference between the VR and control conditions in their average change in dietary footprint from 1 week prior- to 1 week after the intervention. The VR intervention led to a dietary footprint decrease of 20.1 kg CO_2,_ which was significantly larger than the control condition, $b=-\,22.28$, 95% CI $[-\,44.26$, $-\,0.30]$, $t(87)=-\,2.01$, $p=0.047$. Therefore, we accepted Hypothesis 1. The effect size of the reported change in the dietary footprint compared to the change in the control condition was medium *d* = 0.4. As specified in the preregistration plan we investigated the robustness of this finding. The effect of the condition remained significant when dietary lifestyle was accounted for, $b=-\,40.53$, 95% CI $[-\,77.88$, $-\,3.17]$, $t(85)=-\,2.16$, $p=0.034$.

### Immediate impact of the intervention on PEB predictors

**Figure 4:**
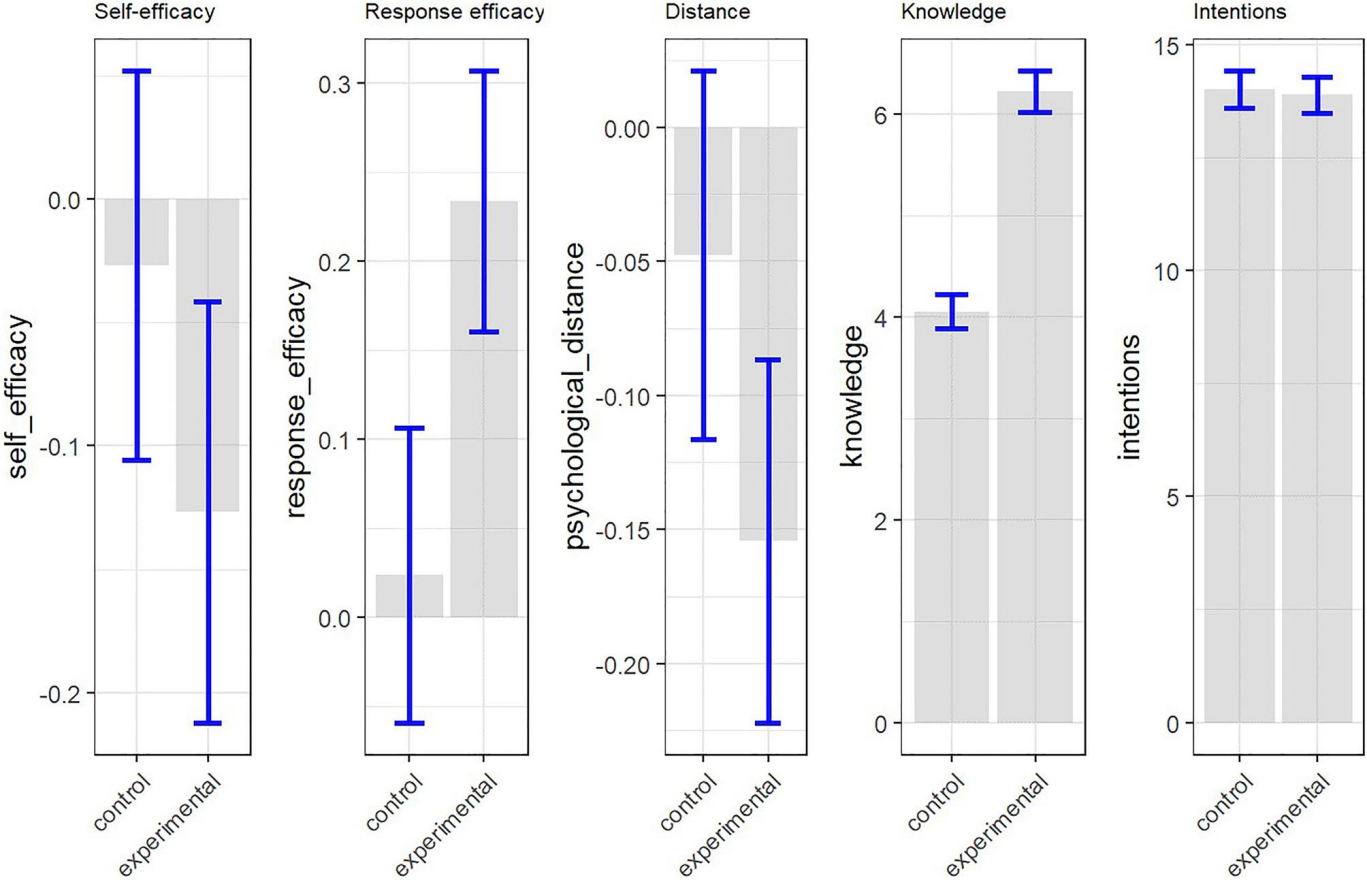
Comparison of the effect of the VR intervention and control condition on the post-treatment measures. All variables are calculated as change scores from baseline to the post-intervention measurement, except for Intentions and Knowledge, which are presented as post-intervention scores. Error bars are 95% confidence intervals.

Furthermore, we compared the impact of the VR and control condition on the identified psychological predictors of pro-environmental behavior: intentions, self-efficacy, response-efficacy, knowledge, and psychological distance measured immediately after the intervention. We found that the VR intervention increased response-efficacy by 0.34 on a 5-point scale, and the increment was larger than in the control condition when controlled for the pre-treatment score, $b=0.20$, 95% CI $[0.00$, $0.40]$, $t(120)=2.02$, $p=0.045$. Similarly, the VR condition resulted in a significantly larger increase in knowledge compared to the control condition when controlled for the preexisting knowledge,$b=2.16$, 95% CI $[1.62$, $2.69]$, $t(120)=7.95$, $p<0.001$. Nevertheless, we did not find a significant difference between the groups on behavioral intentions when controlled for dietary lifestyle, $b=-\,0.01, 95\% CI [-\,0.28, 0.26], t(120)=-\,0.05, p=0.964$, on self-efficacy when controlled for the pretest score, $b=-\,0.02, 95\% CI \left[-\,0.22, 0.18\right], t\left(120\right)=-\,0.21, p=0.835$ or psychological distance when controlled for the pre-intervention score, $b=0.10, 95\% CI [-\,0.08, 0.29], t(120)=1.09, p=0.278.$ Therefore, hypothesis 2 was partially supported (Hypotheses 2C and 2D were confirmed). For a comparison of the effects, see Table 2 and Fig. 4.

**Table 2:** Comparison of the effect of the VR intervention and control condition.

	Control (*n* = 56)	VR Intervention (*n* = 67)	Beta (95% confidence interval)
Dietary footprint	− 5.09 (42.1)	20.1 (79.8)	− 22.28, 95% CI [− 44.26, − 0.30]
Self-efficacy	− 0.03 (0.59)	− 0.13 (0.70)	− 0.02, 95% CI [− 0.22, 0.18]
Response-efficacy	0.02 (0.62)	0.23 (0.60)	0.20, 95% CI [0.00, 0.40]
Psychological distance	− 0.05 (0.51)	− 0.15 (0.55)	0.10, 95% CI [− 0.08, 0.29]
Intentions post	14.0 (3.08)	13.9 (3.33)	− 0.01, 95% CI [− 0.28, 0.26]
Knowledge post	4.05 (1.26)	6.22 (1.69)	2.16, 95% CI [1.62, 2.69]

All variables are calculated as change scores from baseline to the second measurement, except for Intentions and Knowledge, which are presented as post-intervention scores. Beta coefficients report the impact of the VR intervention compared to the control condition when controlled for the pre-intervention scores.

### Lagged impact of the intervention on PEB predictors

Furthermore, as stated in the preregistration analysis plan, we compared the impact of the VR intervention and the control condition on the predictors measured in the one-week follow-up controlling for the pre-intervention scores (or preexisting knowledge in case of knowledge). The results showed that the VR intervention (versus the control group) showed significantly higher knowledge at follow-up when controlled for preexisting knowledge, $b=2.47$, 95% CI $[1.88$, $3.07]$, $t(88)=8.27$, $p<0.001$. Nevertheless, we did not find a significant difference between the conditions on self-efficacy, $t(89)=-\,1.72$, $p=0.088$, response-efficacy, $t(89)=-\,0.85$, $p=0.397$ or psychological distance, $t(89)=-\,0.64$, $p=0.523$. Therefore, only Hypothesis 3C was supported.

### Comparison of the VR conditions

To unpack these findings, we conducted exploratory analyses comparing the impact of the specific VR intervention conditions on the PEB predictors from the posttest (self-efficacy and psychological distance) and on the behavioral outcome (dietary footprint) from the follow-up. Our 2 × 2 design aimed to (1) decrease the psychological distance of the climate change using a geographically proximal (Scandinavia) or distant (USA) location and (2) to increase self-efficacy using normative rather than generic feedback. Both manipulations were focused on enhancing the persuasive impact of the VR intervention and reducing the dietary footprint.

First, we compared the impact of the normative feedback (*n* = 32) on post-intervention self-efficacy to the generic feedback condition (*n* = 35). There was a significant difference in self-efficacy favoring the normative feedback condition compared to the generic feedback condition, $b=0.29$, 95% CI $[0.01$, $0.58]$, $t(63)=2.08$, $p=0.041$. A comparison of the distance conditions did not show any difference in psychological distance between the proximal (*n* = 33) and distant conditions (*n* = 34), $t(63)=0.17$, $p=0.867$. We also did not find any effect of geographical location (*n* = 25), $t(45)=0.41$, $p=0.683$, or normative feedback (*n* = 24) on the reported carbon dietary footprint, $t(45)=0.46$, $p=0.651$.

## Discussions

The results of this randomized trial suggest that an efficacy-focused VR intervention designed according to instructional design guidelines can effectively reduce individual dietary footprints. Compared to the participants in a passive control condition, our VR intervention significantly reduced dietary footprints with a medium effect size (*d* = 0.4). Notably, this finding is robust when controlling for dietary lifestyle and is larger than the typical effect size reported in a previous meta-analysis of behavioral interventions compared to the no-intervention control group to promote household actions mitigating climate change (*d* = 0.093)^46^. Consistent with the intention behind our intervention, which was based on previous theory and research^21,39,35,36^, the findings also support our contention that being able to visualize the consequences of food choices and witness the impact of changing these, in immersive VR can be an effective tool for fostering response-efficacy, which is identified as a crucial predictor of pro-environmental actions^12^.

Contrasting with our predictions, the VR intervention, in general, did not result in a larger increase in self-efficacy beliefs compared to the control condition. However, we did find that the VR intervention had a significantly more positive impact on self-efficacy beliefs when normative feedback was included compared to generic feedback, which is in line with research indicating that relevant normative feedback can increase self-efficacy beliefs^26,47,36^. Our sample reported generally high pretest self-efficacy beliefs (4.2 on a five-point scale), and there is some evidence that initially high self-efficacy can decrease in the first phases of acquiring a new skill^48^. Along these lines, it is plausible that the VR experience results in a revision and correcting of individual beliefs, an idea that is supported by the fact that self-efficacy decreased immediately after the VR intervention for the normative and generic feedback groups, though this change was not significant.

Similarly, and despite the observed changes to individual carbon footprints, we did not find any effect of the VR intervention on reported behavioral intentions. One explanation for this lack of finding is that the items measuring intentions may have been too specific (e.g., “In the future, I intend to refrain from eating meat completely.”) and binding for a climate-aware sample. The low, and non-significant correlation between the observed change in dietary footprint and the measure of intentions (*r* =  − 0.14, *p* = 0.0.16), contrasts with meta-analytic findings of a substantial correlation of *r* = 0.54 between intentions and environmental behavior^49^. This is again suggestive of issues with the measure of intentions. Based on this, we would suggest that future research into the impact of the VR simulations on behavioral intentions ensure that measures of behavioral intentions are valid and plausible within the target population.

Finally, the VR intervention had no impact on the perception of the psychological distance of climate change, and this was not enhanced by the explicit manipulation of distance by locating the simulation somewhere close to (Scandinavia) or far from (USA) participants’ own location. Similarly, the geographical location of the simulation had no bearing on observed changes to dietary footprints. This may have been due to the fact that the proximal condition was still not located within the participants’ own national environment (Denmark), or it could be further evidence that the effects of distance manipulations are not as simple or straightforward as is often assumed^8,30^. Irrespective of this, any findings concerning our manipulations of geographical distance and normative feedback should be considered preliminary in the context of the small cell size for each of the VR conditions.

Our findings regarding the behavioral outcomes are in line with previous VR research showing that VR experience can promote PEB, for example, the virtual experience of cutting or planting a virtual tree reduced the use of paper consumption^39,50^ or experiencing an emotional 360-degree video about meat consumption resulted in selecting more vegetarian food compared to control group^24^. Similarly, our findings are in line with previous studies showing that displaying the impact of the specific food choices on the environment^21^ or mitigating climate change, e.g. by planting a virtual tree^39^ can result in enhanced response efficacy beliefs. Our results also confirmed that experiencing the negative consequences of climate change, e.g. by traveling to places affected by it is an effective way to increase pro-environmental knowledge^20,51^, but contrasting with the previous study we did not find the positive impact of the VR interventions on the environmental intentions compared to the control condition^20^. Contrary to the some^31,32^ but not all VR studies,^57^ varying the level of construal—using more distant or proximal reference, did not result in reduced psychological distance. This missing effect can be an artifact due to underpowered design.

Our findings indicate that VR can be an effective tool for promoting a switch to more plant-based diets and that this effect can be enduring for at least 1 week after the intervention. Furthermore, compared to the previous studies, our outcome measure allows us to calculate the estimated environmental impact in terms of carbon emissions and therefore bridging the discussed motivation-impact gap^52^.

The results of this study should be interpreted based on several additional potential limitations. Firstly, the passive control condition allowed us to control for demand characteristics linked to social desirability bias but not to information bias provided as the participants in the control group did not receive any information. Although some findings indicate that the use of active vs. passive control groups does not result in a significant difference in measured outcomes^53^, future research should focus on comparing the effectiveness of the VR intervention with other methods of environmental communication. Therefore, the results of this randomized trial do not allow us to disentangle the psychological mechanisms that resulted in the reported behavioral change and do not answer the question of what is the additional value of immersive technology compared to more standard interventions. Future research should therefore focus on investigating the cognitive, affective, or educational aspects behind these effects using different comparison conditions including standard knowledge-based interventions or VR emotional embodied experiences focused on connectedness to nature^3^.

Secondly, recruiting participants from a psychology course resulted in a sample with specific demographic characteristics—the majority were young, female university students, resulting in a more climate-aware sample. While previous research shows that climate-aware respondents^54^ are driven to climate action by similar predictors as the general population^12^, that is, efficacy beliefs and social norms, the presented results should be replicated in more diverse samples to improve the generalizability of our findings. Furthermore, future studies should consider environmental anxiety and investigate how people with higher anxiety levels respond to similar VR simulations.

Finally, in this study, we used a self-reported food frequency questionnaire to obtain an estimate of dietary carbon footprint over the course of 1 week^55,36^. Compared to retrospective items measuring general food preferences, the food frequency questionnaire allowed us to capture detailed information and calculate dietary impact in kilograms of carbon. However, this information is still not objective and, despite the controlled design, it could be still influenced by social desirability, as the participants in the VR condition received the necessary factual information and went through an emotional and immersive experience. Future research could attempt to measure spontaneous food choices in the laboratory or observe individual consumption outside of the laboratory setting or use gift vouchers to track participants’ actual shopping choices using gift vouchers to eliminate the risk of desirability bias and the intention-behavior gap^56^.

### Conclusions

The study provides novel evidence for the effectiveness of VR as a tool to promote a switch to more plant-based diets. Moreover, compared to the results of other randomized controlled trials targeting sustainable household behavior, the effect of this intervention is large. Conceptually, these findings support the importance of instructional design principles and a focus on the main drivers of behavioral change when designing VR interventions^8^. Additionally, the fact that the impact of the VR intervention in terms of the carbon footprint, knowledge, and response efficacy was larger than that of the control condition confirms that the effect cannot be reduced to the study’s demand characteristics. Despite the trial limitations, including sample size and specificity, which limit the generalizability of the results, this study has important implications for the future of environmental communication and the possibility to use immersive VR as a potential tool to support the “green transition”. Future research should focus on investigating the psychological mechanisms behind the behavioral results in more detail.

## Supplementary Information

Supplementary Information.

## Data Availability

The datasets generated and/or analyzed during the current study are publicly available in the Open Science Framework repository, https://bit.ly/3C6ZlEO.
