# Supplementary material for: A randomized trial testing the effectiveness of virtual reality as a tool for pro-environmental dietary change
Source: Sci Rep. 2022 Aug 22;12:14315. doi: 10.1038/s41598-022-18241-5 (PMC9395353; doi:10.1038/s41598-022-18241-5)
Supplement: Supplementary file 1 — Supplementary Information. [file 41598_2022_18241_MOESM1_ESM.pdf]

A Randomized Trial Testing the Effectiveness of Virtual Reality as a Tool for Pro-Environmental Dietary Change

Adéla Plechatá[a*], Thomas Morton[a], Federico J.A. Perez-Cueto[b], Guido Makransky[a]

[a] Department of Psychology, University of Copenhagen, Denmark

[b] Department of Food Science, University of Copenhagen, Denmark

1. Supplemental material

1.1 Outcome measures

Dietary lifestyle

What is your dietary lifestyle?

1. *Omnivorous (plant and animal foods)*
2. *Flexitarian*
3. *Pescatarian*
4. *Vegetarian*
5. *Vegan*

Food frequency questionnaire

Please indicate how many times did you eat the following foods during the last week (7 days)? (Never – 8 times or more).

	Never	Once	Twice	3 times	4 times	5 times	6 times	7 times	8 times or more
Beef									
Fish									
Cheese									
Pork									
Poultry									
Pulses/ legumes									
Rice									
Sweets									
Pasta									
Dairy (Milk or Yogurt)									
Fruits									
Vegetables									
Bread									

Response efficacy [17]

- If most people change their eating habits (according to suggested measures) the consequences of climate change will decrease.
- Consumption of food with a low carbon footprint is an effective measure to mitigate climate change.
- If I change my eating habits (according to suggested measures) the consequences of climate change will decrease.

Self-efficacy

- I believe that I have the ability to take action to mitigate global warming and prevent climate change.[45]
- Although it may cause inconvenience, I can still change my behavior to mitigate global warming.[45]
- I feel capable of adopting more climate-friendly eating habits. [17]
- I feel capable of consuming more food with a low carbon footprint. [17]

Intentions [17]

- In the future, I intend to cut the number of meals with meat to half.
- In the future, I intend to refrain from eating meat completely.
- In the future, I intend to replace beef meat with chicken/fish/pork every other meal.
- In the future, I intend to eat vegetarian food twice as often as today.

Psychological distance [36]

- My local area is likely to be affected by climate change.
- Climate change will mostly affect areas that are far away from Denmark.
- Climate change will mostly affect developing countries.

- Climate change is likely to have a big impact on people like me.
- When, if at all, do you think Denmark will start feeling the effects of climate change?

  a) *We are already feeling it*
  b) *Very soon*
  c) *Soon*
  d) *Neutral*
  e) *In distant future*
  f) *In the very distant future*
  g) *Never*

1.2 Additional analyses

Consistent with the main analyses reported in the main text, analyzing the impact of VR intervention on the self-efficacy measured with all four items, we did not find any effect of the VR treatment on the self-efficacy immediately after the intervention, $b = 0.06$, 95% CI [–0.09, 0.22], $t(120) = 0.82$, $p = .413$ or in the follow-up $b =–0.16$, 95% CI [–0.34, 0.03], $t(89)=–1.65$, $p = .102$.

In the exploratory analyses, we also confirmed that when including all self-efficacy items, the normative feedback condition resulted in a significantly larger increase in self-efficacy compared to the no generic feedback, $b = 0.24$, 95% CI [0.02, 0.47], $t(63)=2.14$, $p = .037$.
